# Combined Inhibition of ErbB1/2 and Notch Receptors Effectively Targets Breast Ductal Carcinoma In Situ (DCIS) Stem/Progenitor Cell Activity Regardless of ErbB2 Status

**DOI:** 10.1371/journal.pone.0056840

**Published:** 2013-02-14

**Authors:** Gillian Farnie, Pamela M. Willan, Robert B. Clarke, Nigel J. Bundred

**Affiliations:** 1 Cancer Stem Cell Research, University of Manchester, Institute of Cancer Sciences, Manchester Academic Health Science Centre, Paterson Institute for Cancer Research, The Christie NHS Foundation Trust, Manchester, United Kingdom; 2 Breast Biology Group, University of Manchester, Institute of Cancer Sciences, Manchester Academic Health Science Centre, Paterson Institute for Cancer Research, The Christie NHS Foundation Trust, Manchester, United Kingdom; 3 Academic Department of Surgery, University Hospital of South Manchester NHS Foundation Trust, Wythenshawe Hospital, Education and Research Centre, Manchester, United Kingdom; King's College London, United Kingdom

## Abstract

Pathways involved in DCIS stem and progenitor signalling are poorly understood yet are critical to understand DCIS biology and to develop new therapies. Notch and ErbB1/2 receptor signalling cross talk has been demonstrated in invasive breast cancer, but their role in DCIS stem and progenitor cells has not been investigated. We have utilised 2 DCIS cell lines, MCF10DCIS.com (ErbB2-normal) and SUM225 (ErbB2-overexpressing) and 7 human primary DCIS samples were cultured in 3D matrigel and as mammospheres in the presence, absence or combination of the Notch inhibitor, DAPT, and ErbB1/2 inhibitors, lapatinib or gefitinib. Western blotting was applied to assess downstream signalling. In this study we demonstrate that DAPT reduced acini size and mammosphere formation in MCF10DCIS.com whereas there was no effect in SUM225. Lapatinb reduced acini size and mammosphere formation in SUM225, whereas mammosphere formation and Notch1 activity were increased in MCF10DCIS.com. Combined DAPT/lapatinib treatment was more effective at reducing acini size in both DCIS cell lines. Mammosphere formation in cell lines and human primary DCIS was reduced further by DAPT/lapatinib or DAPT/gefitinib regardless of ErbB2 receptor status. Our pre-clinical human models of DCIS demonstrate that Notch and ErbB1/2 both play a role in DCIS acini growth and stem cell activity. We report for the first time that cross talk between the two pathways in DCIS occurs regardless of ErbB2 receptor status and inhibition of Notch and ErbB1/2 was more efficacious than either alone. These data provide further understanding of DCIS biology and suggest treatment strategies combining Notch and ErbB1/2 inhibitors should be investigated regardless of ErbB2 receptor status.

## Introduction

Ductal carcinoma in situ (DCIS) is a pre-invasive malignant lesion, which if untreated, progresses to invasive cancer in 30–50% of patients [1], [2]. The treatment for DCIS ranges from mastectomy to breast conserving surgery with and without radiotherapy and endocrine therapy [3]. After breast conserving surgery and radiotherapy the DCIS in approximately 15–20% of women recurs within ten years, at which time half the recurrences are invasive disease [2], [4]. There is a need for a more tailored approach to treatment as DCIS, like invasive breast cancer, is a very heterogeneous disease. Evidence suggests that tumours, including breast cancers, may be initiated and maintained by a subpopulation of cells within the heterogeneous tumour. These cells have been shown to have stem cell characteristics and are termed cancer stem cells (CSCs) or tumour initiating cells [5], [6]. CSCs are thought to play a major role in disease recurrence and treatment resistance as both *in vitro* and *in vivo* studies provide evidence of the inherent resistance of breast CSCs to radio and chemotherapy [7]–[9]. In order to target therapeutic strategies and to reduce recurrence and mastectomy rates of DCIS, we need to develop an understanding of the signalling pathways regulating DCIS and CSCs in particular.

We have previously published on the importance of epidermal growth factor receptor (EGFR/ErbB1) signalling, particularly in ErbB2 overexpressing DCIS and also the role for Notch signalling in regulating DCIS cancer stem/progenitor cells [10]. Recent data indicate that in trastuzumab resistant BT474 cells treatment with either trastuzumab or a dual ErbB1/ErbB2 receptor tyrosine kinase inhibitor, 4557W, causes an increase in Notch1 activity. Knockdown of Notch1 using siRNA or reduction of Notch1 signalling using a γ-secretase inhibitor restored trastuzumab sensitivity [11]. Xenograft models of both trastuzumab-sensitive and resistant BT474 ErbB2 positive breast tumours also show that trastuzumab plus a γ-secretase inhibitor (MRK-003) could completely prevent tumour re-growth in sensitive cells after treatment withdrawal and reduce tumour growth in trastuzumab resistant BT474 xenografts [12]. Similar data were reported in basal cell lines (MDA-MB-468 and MDA-MB-231) and a xenograft model of basal-like breast cancer where inhibition of either pathway alone using a γ-secretase or ErbB1 inhibitor had no effect on proliferation or survival, however combination treatment caused a marked increase in cell death and significantly reduced tumour size [13]. The effects seen with combination treatment were in part due to inhibition of AKT activity which could be rescued by re-expressing an active form of Notch1 [13]. An independent study has also highlighted the importance of Notch activated AKT, in which breast epithelial cells over expressing the active form of Notch1 (NICD) showed reduced apoptosis in response to chemotherapy, due to a Notch-induced activation of AKT via an autocrine factor [14].

Cross-talk between ErbB2 and Notch3 has been highlighted in an *in vitro* model of ErbB2 overexpressing DCIS like cells [15]. Transfection of normal MCF-10A cells with ErbB2 produces DCIS like acini structures with filled lumens in matrigel [15], [16] and is associated with up regulation of several components of the Notch pathway including Notch 3 and HES1 [15]. Notch3 siRNA was sufficient to reverse the lumen filled ErbB2 phenotype through induction of apoptosis, indicating that Notch signalling plays a role in the anoikis resistance in ErbB2 overexpressing cells. *In vivo* studies using a MMTV ErbB2/neu transgenic mouse model also confirmed the up regulation of Notch3 in hyperplastic and DCIS like lesions [15]. Individually both Notch and ErbB2 have been shown to highly active or up-regulated in CSC [17]–[19] and play a role in their regulation *in vitro* and *in vivo* [10], [17], [20]. However cross-talk of ErbB and Notch receptor signalling in cancer stem cells is not fully understood.

In the present study, we have used pre-clinical *in vitro* culture models of human DCIS stem and progenitor activity to investigate cross talk between Notch and ErbB1/2. We investigated two DCIS cell lines, MCF10DCIS.com (ErbB2 normal) and SUM225 (ErbB2 overexpressing), and 7 human primary DCIS samples taken from patients after surgery with ErbB2 positive or negative molecular subtypes. We demonstrate significant cross talk between Notch and ErbB receptors in cell lines and primary DCIS samples. CSC activity and acini formation in 3D matrigel of both cell lines and primary DCIS samples was reduced to a greater extent with combination treatment than with either inhibitor alone, regardless of ErbB2 receptor status.

## Results

### Reduction of DCIS acini size and mammosphere formation after Notch or ErbB1/2 inhibition on MCF10DCIS.com and SUM225

**Figure 1:**
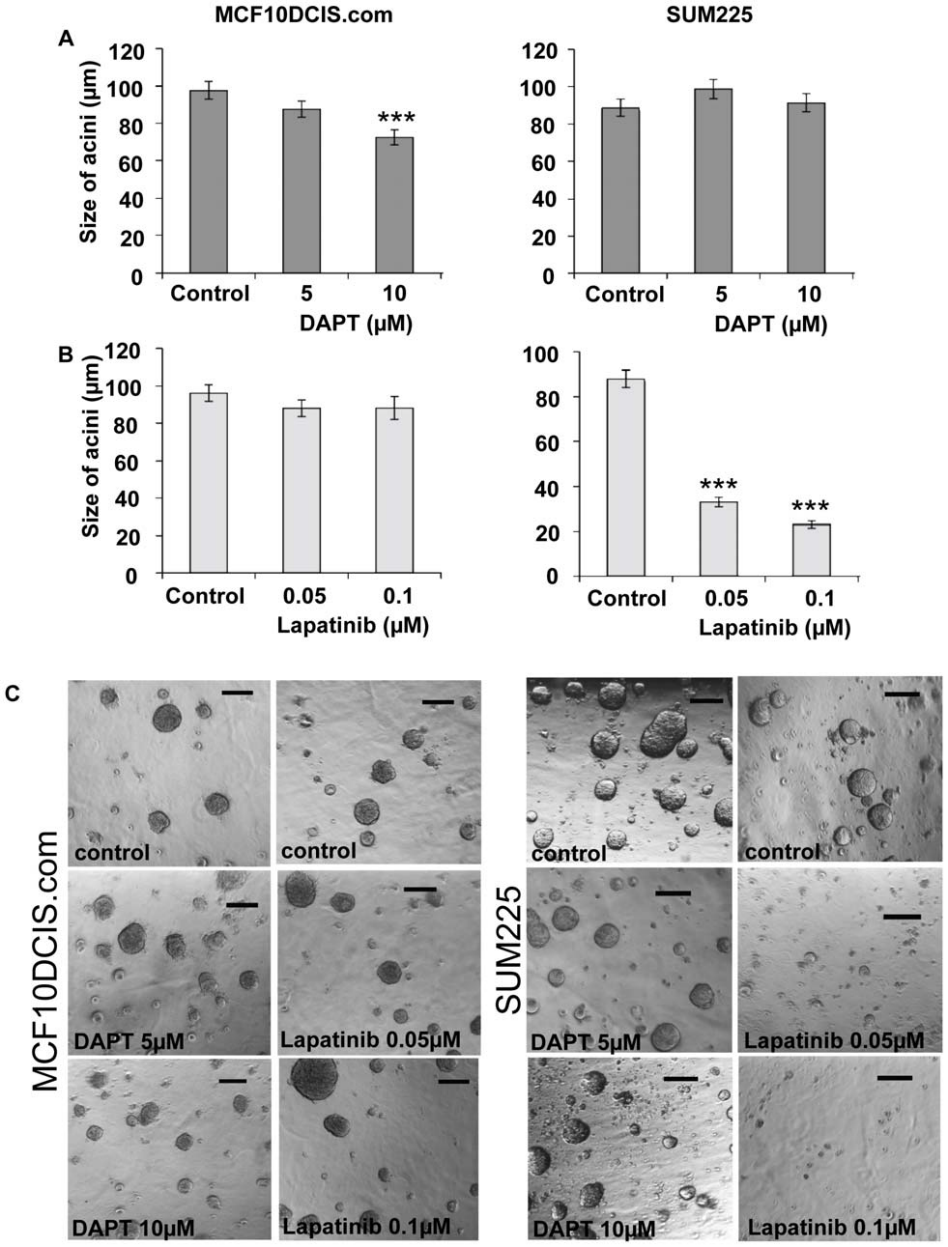
Notch and ErbB1/2 receptor inhibition reduces the size of DCIS cell line acini. MCF10DCIS.com and SUM225 cells were grown in matrigel culture in the presence or absence of DAPT 5 and 10 µM (A), Lapatinib 0.05 and 0.1 µM (B). Cells were treated from day 0 and media/inhibitor was changed every 3 days. After 21 days in culture the size of the acini were measured (µm). (C) Bright field images of DCIS acini grown in the presence or absence of DAPT 5 and 20 µM or Lapatinib 0.05-and 0.1 µM. Scale bar represent 100 µm, graphs represent mean±standard error of 3 independent experiments, Man Witney U test, two-tailed, * p≤0.032, ** p≤0.008, *** p≤0.0001.

To investigate the effects of DAPT or lapatinib on *in vitro* 3D recapitulated DCIS acini MCF10DCIS.com and SUM225 DCIS cell lines were seeded into matrigel culture with increasing doses of inhibitor. This allows the assessment of DCIS cells in a more *in vivo* like environment, facilitating basement membrane interactions that are not present in other *in vitro* culture systems. The number and size of the acini were measured after 21 days of culture. The number of acini did not change after treatment with either inhibitor however the size of DCIS acini was reduced by 25% in the MCF10DCIS.com cells at 10 µM DAPT (p≤0.0001) and no reduction was seen in the SUM225 cell line (Figure 1A&C). Lapatinib treatment caused a 20% decrease in acini size in the MCF10DCIS.com (ErbB2 normal) cell line at 0.5 µM (p = 0.0032) and, as expected, a marked decrease of 62.5% was seen in the SUM225 (ErbB2 overexpressing) cell line at the lowest dose of 0.05 µM (p<0.0001) (Figure 1B&C).

**Figure 2:**
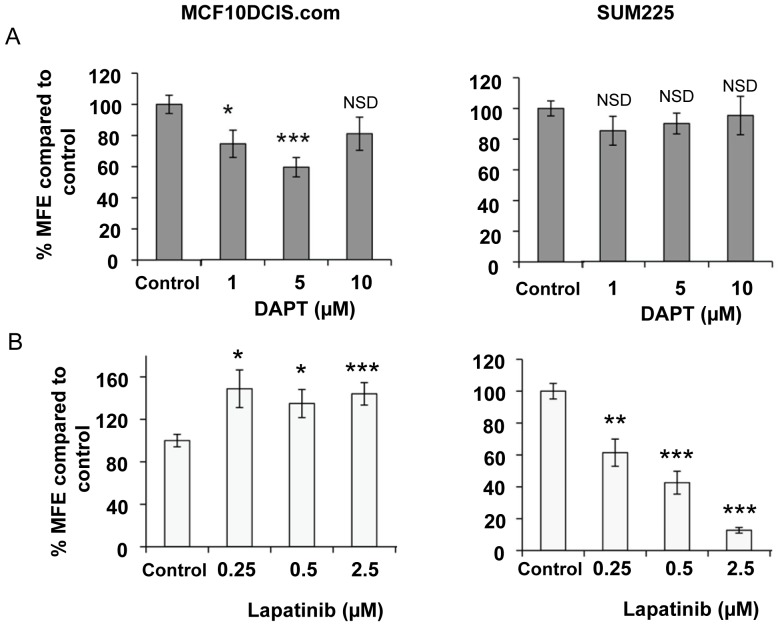
Notch and ErbB1/2 receptor inhibition reduces mammosphere forming efficiency. MCF10DCIS.com and SUM-225 cells were treated with DAPT 1–10 µM (A) or Lapatinib 0.25–2.5 µM (B) in mammosphere culture. Mammosphere-forming efficiency (MFE) was calculated by dividing the number of mammospheres formed by the original number of cells seeded and is expressed as a percentage compared to control. Mean±standard error n = 3, Man Witney U test, two-tailed, * p<0.05, ** p<0.01, *** p<0.001, NSD – No significant difference.

Although the 3D matrigel allows the assessment of the role of these signalling pathways in an *in vivo* like environment the effects are still observed on the bulk of the DCIS cells. To investigate the effects that these inhibitors may have on DCIS cancer stem/progenitor cell activity we have utilised the non-adherent mammosphere assay. DAPT (0.1–10 µM) caused a reduction in MCF10DCIS.com mammosphere formation efficiency (MFE) at 1 µM (p<0.03) and 5 µM (p<0.0003) compared to control but there was no significant effect on the SUM225 MFE at any concentration. In contrast to DAPT, lapatinib caused a marked decrease in MFE in the SUM225 cell line with a 39% reduction with the lowest dose of 0.25 µM (p = 0.01). In the MCF10DCIS.com cell line lapatinib treatment cause an increase in MFE at all doses (0.25–2.5 µM) (Figure 2A and B).

**Figure 3:**
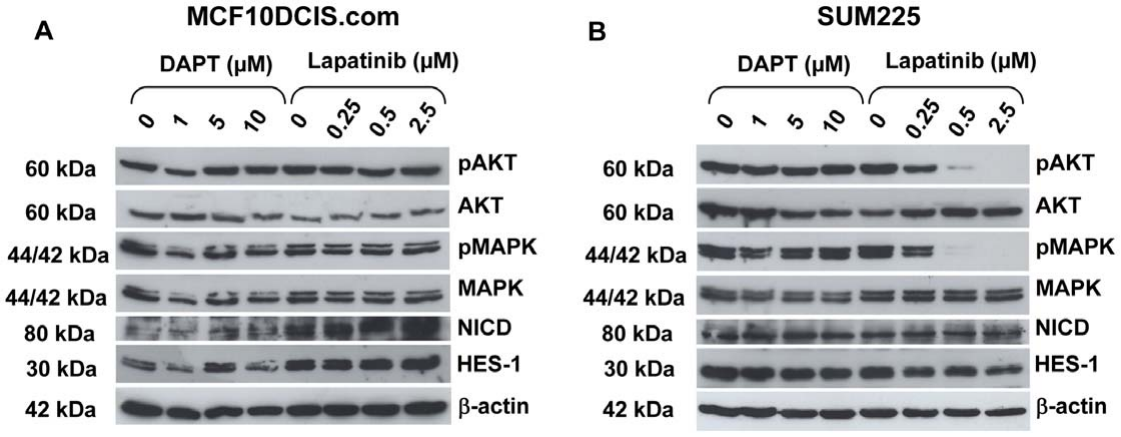
Downstream signalling in DCIS cell line mammospheres. Western blot analysis of downstream targets of Notch and ErbB1/2 receptor signalling in (A) MCF10DCIS.com and (B) SUM225 cells after treatment with DAPT or Lapatinib for 7 days in non-adherent mammosphere culture. Treatments were added at time zero. pAKT  =  phospho-AKT; AKT  =  total AKT; pMAPK  =  phospho-MAPK; MAPK  =  total MAPK; NICD  =  Notch1 intracellular domain. β-actin was used as a loading control.

Changes in signalling within DCIS mammospheres were assessed on control and treated mammospheres after 7 days of non-adherent culture. MCF10DCIS.com mammospheres treated with DAPT showed a decrease in HES1 expression at 1 µM and 10 µM with an unexpected increase at 5 µM but no changes in Notch1 intracellular domain (NICD) (using a cleavage specific Notch 1 antibody), phospho-AKT or phospho-MAPK activity were observed at this time point. Lapatinib treatment on MCF10DCIS.com mammospheres did not alter levels of AKT or MAPK activity but levels of NICD were increased at the 0.5 µM and 2.5 µM dose (Figure 3A). DAPT treatment in the SUM225 caused no decrease in NICD, HES-1, phospho-MAPK or phospho-AKT (Figure 3B, densitometry analysis see Table S1). However, there were marked reductions in phospho-AKT and phospho-MAPK at all concentrations of lapatinib which correlates with the reductions in mammosphere formation (Figure 2B right graph). We also investigated the effects of these inhibitors in ‘bulk’ cells using the same time course in DCIS.com and SUM225 cells in monolayer (data not shown). Under these conditions we saw similar changes overall although Lapatinib at 0.5 µM and 2.5 µM did cause a reduction in pMAPkinase in the MCF10DICS.com unlike the day 7 mammosphere cultures where no reduction was seen (Figure 3A).

### Combined Notch and ErbB1/2 inhibition reduces DCIS acini size and mammosphere formation regardless of ErbB2 status

**Figure 4:**
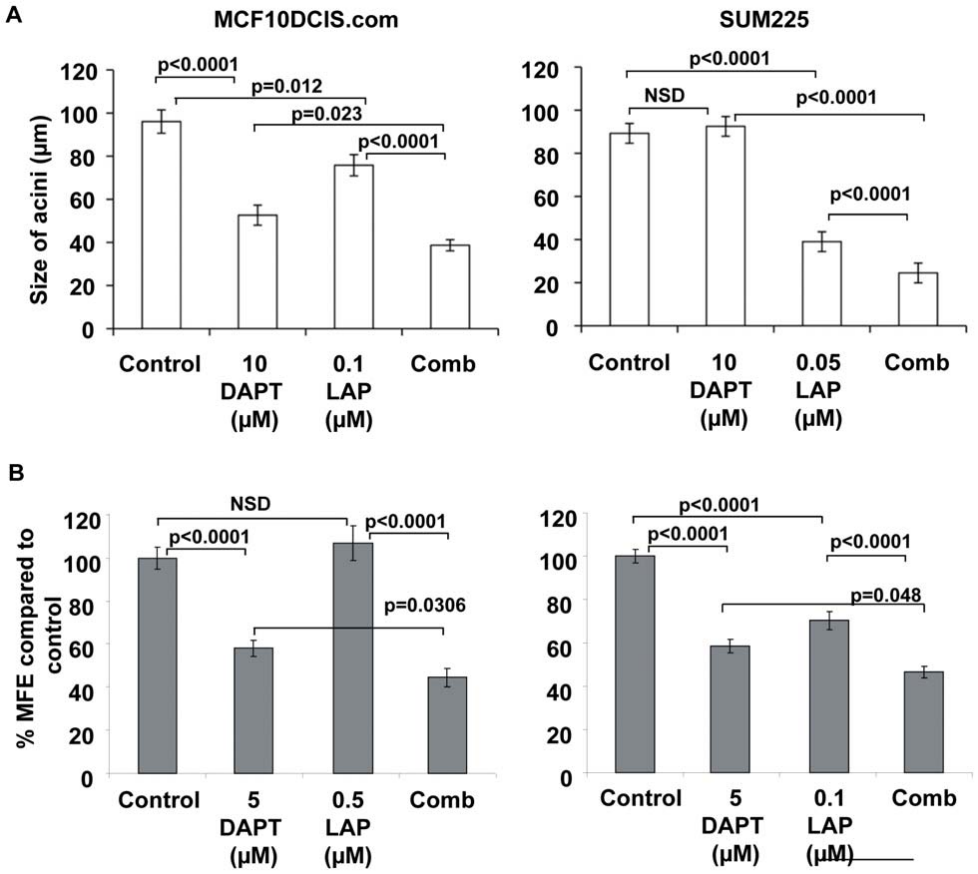
The combination of Notch and ErbB1/2 receptor inhibition reduces acinar size and mammosphere formation regardless of ErbB2 status. MCF10DCIS.com and SUM225 cells were treated with control, DAPT, lapatinib or combination (comb) in (A) matrigel culture and (B) mammosphere culture. (A) Cells were treated from day 0 and media/inhibitor were changed every 3 days. After 21 days in culture the sizes of the acini were measured ( µm).(B) Mammosphere-forming efficiency (MFE) was calculated by dividing the number of mammospheres formed by the original number of cells seeded and is expressed as a percentage compared to control. Mean±standard error of 3 independent experiments, Man Witney U test, two-tailed, NSD – No significant difference.

The concentration of DAPT and Lapatinib used within the combination experiments differed both between cell lines and the type of in vitro assay, matrigel/acini or mammosphere culture. Only concentrations causing ≤40% reduction in acini size or mammosphere formation were used in order to fully explore any further reduction with combination treatment. In MCF10DCIS.com cells, combined treatment caused a further reduction in acini size of 27% (p = 0.023) and 50% (p<0.0001) compared to DAPT 10 µM (p<0.001) or 0.1 µM lapatinib (p = 0.012) treatment alone respectively. Within this experiment addition of 0.1 µM lapatinib caused a significant reduction in acini formation which was not observed in Figure 1B, but this did not affect the additional reduction seen by the combination treatment. The effect of the combination in the SUM225 cell line also yielded additional reductions of 74% (p<0.0001) and 38% (p<0.0001) when compared to either 10 µM DAPT (NSD) or 0.05 µM Lapatinib (p = <0.001) alone respectively (Figure 4A).

The mammosphere assay revealed that the combination of DAPT 5 µM (p<0.0001) and lapatinib 0.5 µM (NSD) in the MCF10DCIS.coms was more effective at reducing MFE than either inhibitor alone, showing a further reduction of 58% (p = 0.001) and 77% (p<0.0001) respectively (Figure 4B). Combined treatment in the SUM225 also resulted in a significant reduction in MFE showing a further 20% (p = 0.0048) and 34% (p<0.0001) reduction compared to the MFE for DAPT 5 µM (p<0.001) or lapatinib 0.1 µM (p<0.001) treatment alone (Figure 4B). These data suggest combination of both inhibitors was more effective at reducing acini size and DCIS stem and progenitor cell activity regardless of ErbB2 receptor status.

### Combination of Notch and ErbB1/2 inhibition in primary DCIS mammosphere formation is better than either inhibitor alone

**Figure 5:**
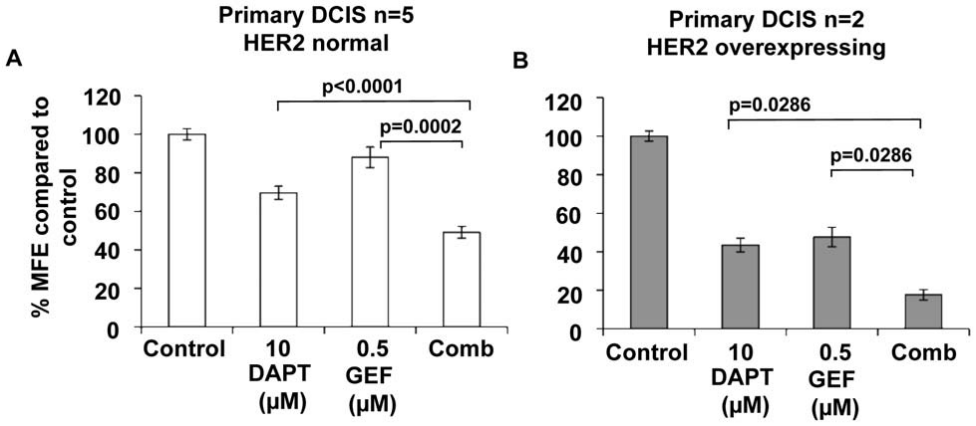
Mammosphere forming efficiency in samples of human primary DCIS is reduced by combined Notch and ErbB1/2 receptor inhibition regardless of ErbB2 status. Primary DCIS cells from 7 patients were grown as mammospheres over 3 days and were treated with control, DAPT 10 µM, gefitinib 0.5 µM or a combination (comb - DAPT 10 µM and gefitinib 0.5 µM). Graphs show DCIS stratified by their ErbB2 receptor status, ErbB2 negative (A) and ErbB2 positive (B). Mammosphere-forming efficiency (MFE) was calculated by dividing the number of mammospheres formed by the original number of cells seeded and is expressed as a percentage compared to control. Mean±standard error, Man Witney U test, two-tailed test.

We validated the cell line data in human primary DCIS samples freshly isolated from patients after surgery. Figure 5 shows the mammosphere culture of 7 DCIS samples, processed as described in [10]. Primary DCIS samples were stratified into ErbB2 normal (n = 5, Figure 5A) and ErbB2 overexpressing (n = 2, Figure 5B). DAPT 10 µM alone caused a 31% (p<0.0001) reduction in MFE compared to control, however gefitinib 0.5 µM alone had no significant effect. Combination of both inhibitors was more effective that either inhibitor alone within the ErbB2 normal DCIS samples, as previously shown for the MCF10DCIS.com cell line. Compared to DAPT or gefitinib treatment alone, combined treatment reduced MFE by 20% (p = 0.0002) and 39% (p<0.0001) respectively (Figure 5A). In ErbB2 overexpressing DCIS samples gefitinib (0.5 µM) treatment decreased MFE by 53% (p = 0.0286) compared to control and unlike the SUM225 (ErbB2 overexpressing) cell line, DAPT (10 µM) also caused a reduction in MFE of 57% (p = 0.0286) compared to control. However, a greater reduction in MFE of 26% (p = 0.00286) and 30% (p = 0.00286) was seen in the combined treatment compared to DAPT 10 µM or gefitinib 0.5 µM alone (Figure 5B). These data demonstrate that combination treatment is more effective at reducing primary DCIS mammosphere formation, regardless of ErbB2 receptor status

## Discussion

We have established useful *in vitro* culture models of human DCIS to investigate signalling pathways that regulate DCIS CSC activity and acini growth in 3D matrigel culture. We use these DCIS models to establish that combined inhibition of both Notch and ErbB receptor pathways reduces CSC activity and acini growth in 3D matrigel culture regardless of ErbB2 status.

In the ErbB2 normal MCF10DCIS.com cell line, the Notch inhibitor, DAPT, caused a reduction in both acinar size and mammosphere formation (Figure 1A and 2A). This effect was not seen in the ErbB2 overexpressing SUM225 cell line, where no significant differences were seen in mammosphere formation compared to control and only a small reduction was seen in acini size at the highest dose of DAPT. These data indicate that SUM225 growth is as not dependent on Notch signalling. This is consistent with the finding that other ErbB2 overexpressing breast cancer cells, SKBR3, BT474 and MCF7/HER-18 have low Notch transcriptional activity as measured by NICD binding to CBF-1 responsive elements in a luciferase reporter [11]. However Western blotting did not indicate a marked reduction in the cleaved form of Notch1 or the down stream target Hes-1 in Sum225 mammospheres compared to the DCIS.com mammospheres (Figure 3). Although other active Notch receptors or downstream targets such as Hey-1 or Hey-L may have been altered or the investigation of Hes-1 levels after a 7 day time course may not be optimal within the SUM225 cell line.

The effect of the ErbB1/2 inhibitor lapatinib was also measured in both culture methods and, as expected, only the ErbB2 overexpressing SUM225 cell line showed a marked decrease in both acini size and mammosphere formation. Interestingly in the ErbB2 normal MCF10DCIS.com cell line, lapatinib treatment significantly increased mammosphere formation compared to control. Analysis of mammospheres at Day 7 showed that expression of the cleaved form of Notch1 (NICD) were elevated (Figure 3) which may explain the increase in stem cell activity observed. Increased full length Notch1 has been reported in HCC-1896 and MDA MB 231 (ErbB2 normal) cells after treatment with the ErbB1/2 inhibitor, gefitinib [13] and ErbB2 inhibition caused increased Notch1 receptor activity in ErbB2 overexpressing cell lines [11]. These data and our own suggest cross talk between the signalling pathways, although further experimental evidence would be needed to verity these findings in the DCIS model.

However, our study demonstrates cross talk between Notch and ErbB2 in DCIS cell lines regardless of ErbB2 receptor status. The combination of DAPT and lapatinib showed an additive reduction in both size of acini and mammosphere formation in the MCF10DCIS.com and the SUM225 cells (Figure 4). Since there are no other DCIS cell lines commercially available to investigate this finding, it was essential to verify these results using human DCIS samples acquired after surgery. The dissociated human primary DCIS cells were grown as mammospheres in identical culture conditions to the DCIS cell lines and the results confirmed that the combination of Notch and the EGFR (ErbB1) inhibitor, gefitinib was more effective that either alone (Figure 5). The mechanism of the additive effect is not clear but our data suggest that within the ErbB2 normal DCIS samples the additional efficacy may be due to an up regulation in Notch1 activity, leading to a more effective inhibition when both ErbB1 and Notch inhibitors are used. Gefitinib specifically inhibits EGFR which is one of the preferred binding partners of ErbB2. This allows not only the inhibition of EGFR homodimers but also EGFR/ErbB2 hetrodimers. Therefore EGFR inactivation via gefitinib within the patient DCIS also suggests EGFR may be important particularly in the wild type ErbB2 samples where the combination treatment caused a greater reduction on mammosphere formation. The mechanism of inhibition in the ErbB2 overexpressing cell line remained unidentified since no changes in signalling pathways were shown after treatment with either drug after 7 days in mammosphere culture. The effects of both inhibitors seen in the DCIS cell lines and primary samples may be due to effects on proliferation, self-renewal or apoptosis. Other studies suggest a reduction in cell survival after combined treatment with Notch and ErbB2 inhibitors [11], [13] via a reduction in AKT activity and this is consistent with our findings.

Our results provide the first evidence that cross talk between ErbB1/2 and Notch pathways is present in DCIS regardless of ErbB2 receptor status. We demonstrate this using *in vitro* models of DCIS including human primary samples to investigate DCIS cell signalling. CSC activity and acini formation of both cell lines and primary DCIS samples was reduced to a greater extent with combination treatment than with either inhibitor alone, regardless of ErbB2 receptor status. These data provide further understanding signalling pathways involved in regulation of DCIS stem and progenitors. We conclude that treatment strategies combining both Notch and ErbB1/2 receptor inhibitors should be investigated in DCIS regardless of ErbB2 receptor status.

## Materials and Methods

### Cell culture and reagents

Human DCIS cell lines (purchased from Asterand, 2007) SUM225 (ErbB-2 overexpressing) and MCF10DCIS.com (ErbB-2 normal) were grown in adherent culture in their relevant medium, MCF10DCIS.com cells were grown in advanced DMEM/F12, 5% (v/v) horse serum, L-glutamine 50 µg/ml all purchased from Gibco. The SUM225 cells were grown in Ham's F12 (Gibco), insulin 5 µg/ml, hydrocortisone 1 µg/ml, HEPES 10 mM and 5% (v/v) foetal calf serum. All cells were maintained in a humidified incubator at 37°C at an atmospheric pressure of 5% (v/v) carbon dioxide/air. The Notch inhibitor DAPT was purchased from Calbiochem, gefitinib was a gift from Astra Zeneca and lapatinib was a gift from GlaxoSmithKline: all were dissolved in 100% DMSO to a 10 mM stock solution.

### Patient samples

Women were included in the study if they had mammograms showing widespread microcalcification indicative of DCIS (n = 7) and histopathological confirmation of the diagnosis of DCIS. All tissue samples were obtained following therapeutic DCIS surgery and were subsequently reviewed and graded by a consultant breast pathologist. All patients provided written consent and the approval to remove tissue from pathological samples was granted by the South Manchester Medical Research Ethics Committee (LREC 01/012).

### Digestion of primary DCIS tissue for mammosphere culture

DCIS tissue was prepared as previously described [10]. Briefly, DCIS tissue collected at surgery was dissected and enzymatically digested overnight (16–18 h) at 37°C in serum free DMEM (Gibco) containing type I collagenase 200 U/ml (Worthington) and 5% penicillin/streptomycin (Sigma). The digest was then filtered to obtain a single cell suspension. Cells were then washed 3 times by centrifugation at 1000 g in DMEM:F12 medium (Gibco).

### Mammosphere Culture

All experiments using cell lines or primary samples were seeded at 500 cells/cm^2^ in appropriate polyHEMA (Poly (2-hydroxyethyl methacrylate) coated tissue culture plates or flasks (Corning). Mammosphere culture medium comprised DMEM-F12 media (Gibco) supplemented with B27 without vitamin A (Gibco) and Epidermal Growth Factor (EGF) 20 ng/ml (Sigma). Mammospheres were counted when their diameter was ≥60 µm. Mammosphere forming efficiency (MFE) was calculated as the percentage of MS formed relative to the original number of single cells seeded (Mean% MFE ± standard error [SEM])

Treatments were added to DCIS cell suspensions at day 0. Treatments included; gefitinib (an EGFR tyrosine kinase inhibitor), DAPT– IX (a γ-secretase inhibitor. Calbiochem) and lapatinib (a HER2/EGFR tyrosine kinase inhibitor) using DMSO control. MS were counted at day 5 or day 3 for the DCIS cell lines and primary samples respectively and expressed as percentage MS formation compared to a non-treated control.

### Matrigel culture

Single cell suspensions of DCIS cell lines were seeded at a density of 5000 cells/well in the appropriate adherent culture medium containing 2% growth factor-reduced matrigel into 8-well glass chamber slides containing 50 µl growth factor reduced matrigel (BD Bioscience). Inhibitors were added to the matrigel media from day 0, DAPT and Lapatinib were used alone and in combination as well as a DMSO control. The cells produced acini over a 21-day period, during which the growth medium (with or with our inhibitor/s) was replaced every 2–3 days. Acini were then counted and sized under a light microscope using an eyepiece graticule with crossed scales.

### Western Blotting

Lysates from cells containing 50 µg of protein were fractionated by SDS-PAGE and transferred to Hybond nitrocellulose membrane (Amersham). The nitrocellulose was then blocked in Tris-buffered saline (TBS) containing 0.1% Tween-20 and 5% nonfat milk for 1 h at room temperature. The membrane was then incubated with primary antibody for 1 h at room temperature or 4°C overnight with gentle shaking. The membrane was then washed three times in TBS containing 0.1% Tween 20 and then incubated with horseradish peroxidase conjugated secondary antibody (1∶5000; Dako) for 1 h at room temperature. After washing, immunoreactive proteins were detected by enhanced chemiluminescence (Pierce). Primary antibodies for Western blotting were:- AKT (cell signalling) 1∶1000, Phospho AKT (Ser 473) (cell signalling) 1∶1000*, ERK (cell signalling) 1∶1000, pERK (cell signalling) 1∶1000*, Anti-human NOTCH 1 cleaved N terminal Cat#100-401-407 (Only the cleaved intracellular (activated) form (NICD) is detected), Rocklands) 1∶500*, Hes-1 (Calbiochem) 1∶1000, β-Actin (sigma), (*overnight incubation at 4°C). MacBiophotonics Image J (1.42l) was used to quantitate protein bands.

## Supporting Information

Table S1 Densitometry analysis of protein levels within Figure 3.
(DOC)
