# Supplementary material for: Combined Inhibition of ErbB1/2 and Notch Receptors Effectively Targets Breast Ductal Carcinoma In Situ (DCIS) Stem/Progenitor Cell Activity Regardless of ErbB2 Status
Source: PLoS One. 2013 Feb 14;8(2):e56840. doi: 10.1371/journal.pone.0056840 (PMC3572946; doi:10.1371/journal.pone.0056840)
Supplement: Table S1 — Densitometry analysis of protein levels within Figure 3. (DOC) [file pone.0056840.s001.doc]

|  | **MCFDCIS.com** | | | | | | | | **SUM225** | | | | | | | |
| --- | --- | --- | --- | --- | --- | --- | --- | --- | --- | --- | --- | --- | --- | --- | --- | --- |
|  | **DAPT (µM)** | | | | **Lapatinib (µM)** | | | | **DAPT (µM)** | | | | **Lapatinib (µM)** | | | |
|  | **0** | **1** | **5** | **10** | **0** | **0.25** | **0.5** | **2.5** | **0** | **1** | **5** | **10** | **0** | **0.25** | **0.5** | **2.5** |
| **pAKT** | 1 | 0.67 | 1.09 | 1.15 | 1 | 0.85 | 0.78 | 0.905 | 1 | 1.2 | 1.38 | 1.79 | 1 | 0.53 | 0.05 | 0 |
| **pMAPK** | 1 | 1.09 | 0.99 | 0.90 | 1 | 1.06 | 1.00 | 0.95 | 1 | 0.83 | 1.1 | 1.41 | 1 | 0.65 | 0.03 | 0 |
| **NICD** | 1 | 0.67 | 1.49 | 1.3 | 1 | 1.04 | 1.06 | 1.59 | 1 | 1.06 | 1.27 | 1 | 1 | 1.03 | 1.12 | 1.38 |
| **HES-1** | 1 | 0.84 | 1.71 | 0.9 | 1 | 0.98 | 0.96 | 1.08 | 1 | 0.91 | 0.99 | 0.8 | 1 | 0.82 | 1.02 | 0.79 |

**Table S1 – Densitometry analysis of protein levels within Figure 3**

Densitometry was performed on Western blots within Figure 3 using MacBiophotonics Image J (1.42l). All bands were all normalised with respect to the β-actin loading control and then fold change was calculated from the control (0µM) for each cell line and each inhibitor. In the case of pAKT and pMAPK these were also normalised with the respective total AKT or MAPK controls.

pAKT = phospho-AKT; AKT = total AKT; pMAPK = phospho-MAPK; MAPK = total MAPK; NICD = Notch1 intracellular domain.
